# Conditional Forest Models Built Using Metagenomic Data Accurately Predicted Salmonella Contamination in Northeastern Streams

**DOI:** 10.1128/spectrum.00381-23

**Published:** 2023-03-22

**Authors:** Taejung Chung, Runan Yan, Daniel L. Weller, Jasna Kovac

**Affiliations:** a Department of Food Science, The Pennsylvania State University, University Park, Pennsylvania, USA; b Microbiome Center, Huck Institutes of the Life Sciences, The Pennsylvania State University, University Park, Pennsylvania, USA; c Department of Statistics and Computational Biology, University of Rochester Medical Center, Rochester, New York, USA; University of Minnesota Twin Cities

**Keywords:** surface water, *Salmonella*, water safety, microbiome, machine learning, indicators

## Abstract

The use of water contaminated with Salmonella for produce production contributes to foodborne disease burden. To reduce human health risks, there is a need for novel, targeted approaches for assessing the pathogen status of agricultural water. We investigated the utility of water microbiome data for predicting Salmonella contamination of streams used to source water for produce production. Grab samples were collected from 60 New York streams in 2018 and tested for Salmonella. Separately, DNA was extracted from the samples and used for Illumina shotgun metagenomic sequencing. Reads were trimmed and used to assign taxonomy with Kraken2. Conditional forest (CF), regularized random forest (RRF), and support vector machine (SVM) models were implemented to predict Salmonella contamination. Model performance was assessed using 10-fold cross-validation repeated 10 times to quantify area under the curve (AUC) and Kappa score. CF models outperformed the other two algorithms based on AUC (0.86, CF; 0.81, RRF; 0.65, SVM) and Kappa score (0.53, CF; 0.41, RRF; 0.12, SVM). The taxa that were most informative for accurately predicting Salmonella contamination based on CF were compared to taxa identified by ALDEx2 as being differentially abundant between Salmonella-positive and -negative samples. CF and differential abundance tests both identified Aeromonas salmonicida (variable importance [VI] = 0.012) and *Aeromonas* sp. strain CA23 (VI = 0.025) as the two most informative taxa for predicting Salmonella contamination. Our findings suggest that microbiome-based models may provide an alternative to or complement existing water monitoring strategies. Similarly, the informative taxa identified in this study warrant further investigation as potential indicators of Salmonella contamination of agricultural water.

**IMPORTANCE** Understanding the associations between surface water microbiome composition and the presence of foodborne pathogens, such as Salmonella, can facilitate the identification of novel indicators of Salmonella contamination. This study assessed the utility of microbiome data and three machine learning algorithms for predicting Salmonella contamination of Northeastern streams. The research reported here both expanded the knowledge on the microbiome composition of surface waters and identified putative novel indicators (i.e., *Aeromonas* species) for Salmonella in Northeastern streams. These putative indicators warrant further research to assess whether they are consistent indicators of Salmonella contamination across regions, waterways, and years not represented in the data set used in this study. Validated indicators identified using microbiome data may be used as targets in the development of rapid (e.g., PCR-based) detection assays for the assessment of microbial safety of agricultural surface waters.

## INTRODUCTION

According to the U.S. Centers for Disease Control and Prevention (CDC), 46% of foodborne illnesses reported in the United States between 1998 and 2008 with a known food vehicle were linked to fresh produce consumption (1). In the United States, Salmonella is the most common bacterial pathogen associated with outbreaks linked to fresh produce (2, 3). Thus, preventing Salmonella contamination of fresh produce is critical for managing foodborne disease burden in the United States.

Multiple produce-associated outbreaks have been putatively traced back to the use of contaminated water for produce production (4–7). Therefore, identifying when water is likely to be contaminated by foodborne pathogens is a central component of preharvest produce safety risk management plans. In many countries, Escherichia coli-based standards have been established for agricultural and recreational water (8–11). However, E. coli is an indicator of fecal and not pathogen contamination. Indeed, the presence and direction of the association between E. coli levels and foodborne pathogen presence vary substantially within the scientific literature, with some studies reporting positive relationships (12–16) and others reporting negative or no relationships (17–20). As a result, E. coli appears to be an unreliable indicator of pathogen contamination of surface waterways, even though E. coli can be used as a general indicator of the hygienic condition of water (17, 21). Thus, there is a need for novel approaches for identifying when and where agricultural waterways may be contaminated with foodborne pathogens, such as Salmonella.

Metagenomics opened new avenues for characterization of water microbiomes. Concurrent characterization of microbiome and pathogen status in water provides an opportunity for the identification of microbial taxa associated with pathogen contamination of agricultural water. Such taxa could be identified by developing models that use microbiome data (e.g., presence or absence of taxa and differences in their relative abundance) to predict when and where pathogens are likely to be present. However, since the existing water microbiome literature demonstrates substantial spatial and temporal variation in water microbiome composition (22–25), identification of such “indicator” taxa is difficult using conventional analytical approaches, such as multivariate ordination (25). Machine learning provides an alternative approach that may be useful for identifying indicator taxa or combinations of indicator taxa and for developing models that use these taxa to predict pathogen contamination status (26, 27). Identified indicators can then be used as targets in the development of rapid (e.g., PCR-based) detection assays for the assessment of microbial safety of agricultural surface waters.

A variety of supervised classification models have been developed to address different data structure challenges and improve prediction accuracy (28–31). A benchmarking study that compared the performance of multiple machine learning algorithms for microbiome-based disease prediction (i.e., Crohn’s disease, ulcerative colitis, ischemic colitis, and obesity) found that model performance varied substantially between algorithms (28). This suggests the importance of selecting and testing multiple algorithms to improve prediction accuracy. Model performance can also be affected by microbiome data preprocessing, such as data normalization (28). The latter is commonly carried out to account for potential differences in sample library sizes; thus, its effect on prediction accuracy needs to be assessed (31–33).

With these considerations in mind, we applied multiple machine learning classifiers to normalized and nonnormalized microbiome data representing 60 samples, each collected from a separate Northeastern stream. Our goal was to identify putative indicators for Salmonella contamination of Northeastern streams that provide water used for produce production. We also assessed whether including environmental data as predictors in the machine learning model increased prediction accuracy.

## RESULTS

### Samples were sequenced with a median of 5,956,185 reads, and a median of 8.955% of reads were assigned a bacterial taxonomic identifier.

A median of 5,956,185 reads per sample were obtained from 60 samples (minimum [min] = 4,048,684, maximum [max] = 9,301,059, standard deviation [SD] = 1,125,759), and a median of 8.95% of reads were classified as bacterial using metagenomics taxonomic classifier Kraken2 (median = 529,963, min = 145,211, max = 1,059,311, standard deviation [SD] = 206,336). Across samples, a total of 2,557 different species and 885 different genera from 307 different families were assigned.

### Overall microbiome composition was not associated with Salmonella presence in surface water samples.

**FIG 1:**
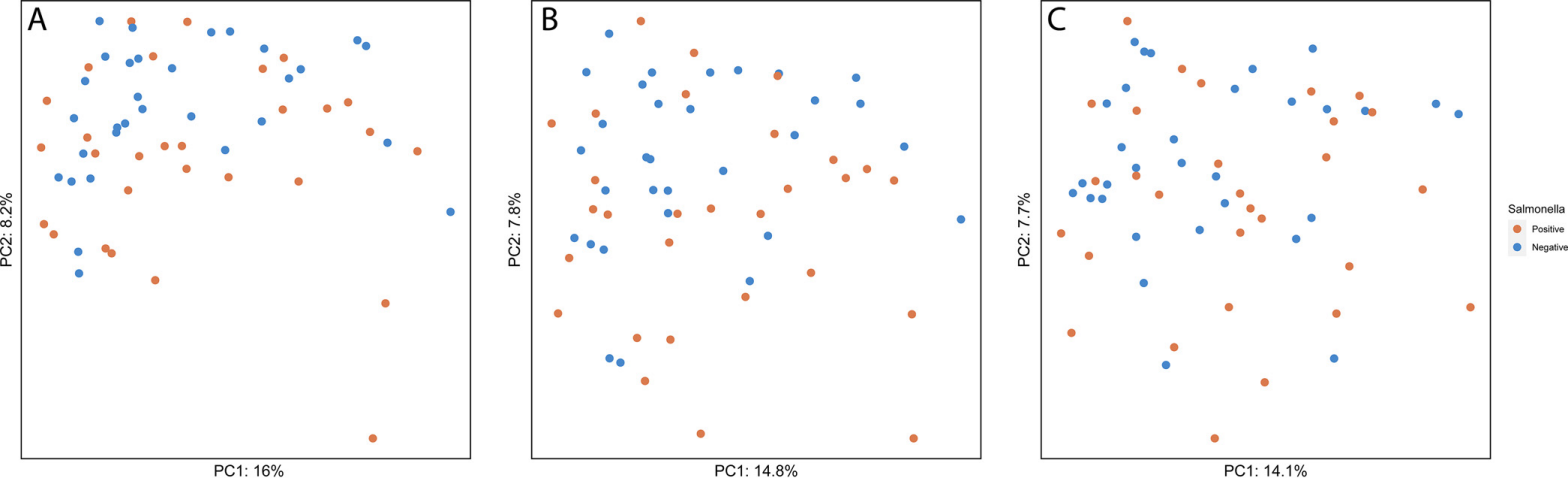
Principal-component analysis (PCA) based on the Aitchison distance. The PCA biplot showing ordination of samples based on the microbiome composition at the species level (A), genus level (B), and family level (C) and color coded based on whether Salmonella was detected (orange) or not detected (blue) in water samples.

The overall microbiome composition was not associated with Salmonella contamination (see Fig. S1 in the supplemental material). Consistent with results of permutational multivariate analysis of variance (PERMANOVA), the principal-component analysis (PCA) biplot (Fig. 1) showed that the sample microbiomes did not cluster based on the presence of Salmonella. The first two components explained a relatively low percentage of variance in the microbiome composition. Specifically, they explained 24.2% of the variance at the species level (Fig. 1A), 22.6% of the variance at the genus level (Fig. 1B), and 21.8% of the variance at the family level (Fig. 1C). PERMANOVA results also did not indicate significant association between microbiome composition and Salmonella isolation (*P* = 0.091 [species level], *P* = 0.349 [genus level], *P* = 0.318 [family level]).

### CF models outperformed RRF and SVM models when predicting Salmonella contamination.

**FIG 2:**
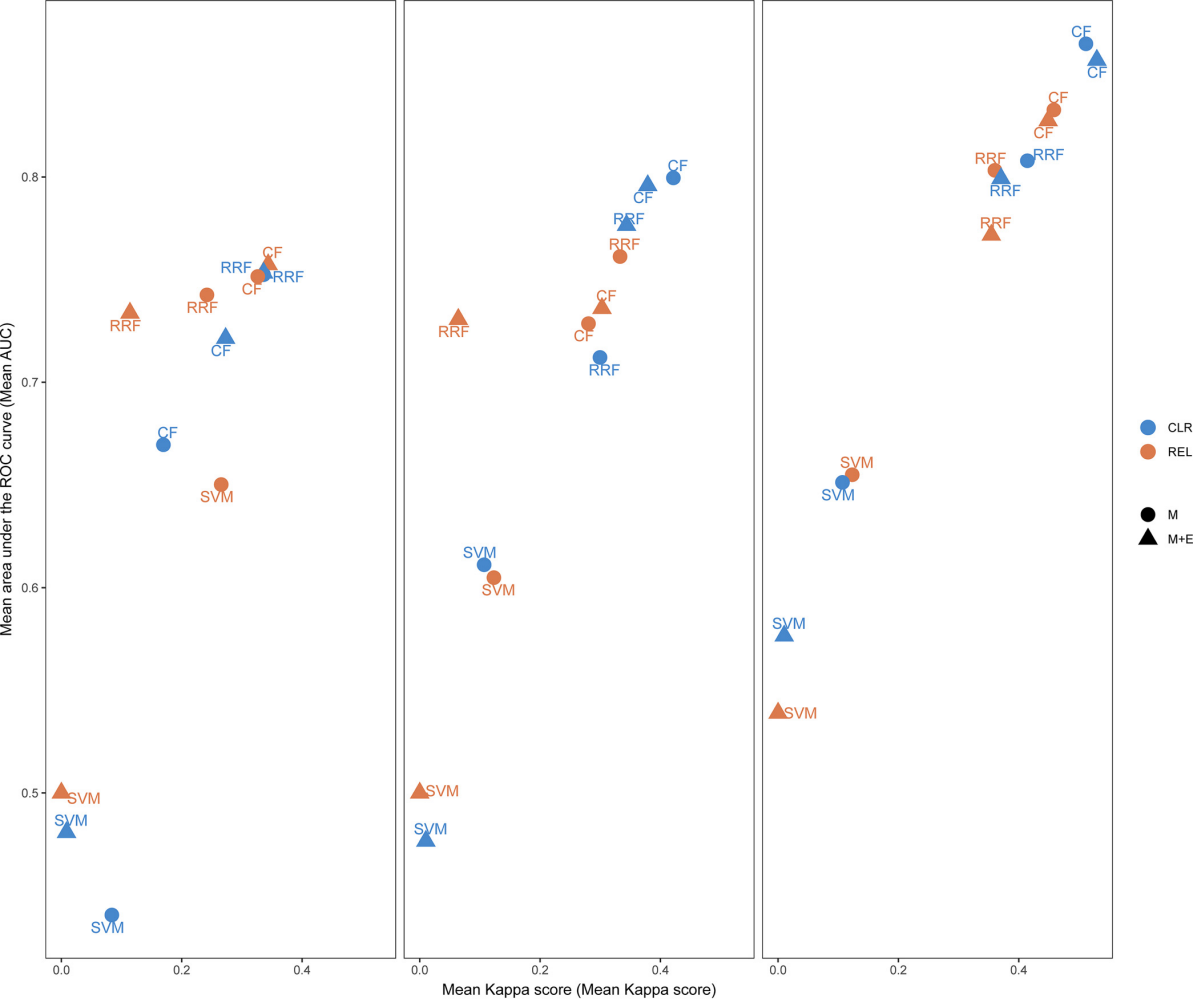
Mean Kappa score (*x* axis) and mean area under the curve (AUC) (*y* axis) for each machine learning algorithm from 10-fold repeated cross-validation. Results are shown at three taxonomic levels used for the classification (left, family; middle, genus; right, species). Two different data transformation methods (centered log-ratio transformation [CLR] [blue] and relative abundance [REL] [orange]) were compared with two different data structures: microbiome data only (M) (circles) and microbiome and environmental data (M+E) (triangles). ROC, receiver operating characteristic.

Regardless of the feature set, the mean area under the curve (AUC) and Kappa score were always higher for conditional forest (CF) than for regularized random forest (RRF) and support vector machine (SVM) when species-level microbiome data were used (Fig. 2; Table S2). The AUC was also consistently higher for CF models than for RRF and SVM models when genus-level and family-level microbiome data were used. The Kappa values were similar for CF and RRF models when genus-level and family-level microbiome data were used (Fig. 2). RRF models (AUC = [0.71, 0.81], Kappa = [0.41, 0.06]) and SVM models (AUC = [0.44, 0.65], Kappa = [0.01, 0.12]) had lower AUCs and Kappa scores than CF models (AUC = [0.67, 0.86], Kappa = [0.17, 0.53]). We found that the AUC range for CF did not overlap AUC ranges of the other two methods, indicating that CF has outperformed RRF and SVM. Hence, further analyses were carried out using the CF models only.

Across all models built using the species-level data, the CF model run on central log-ratio (CLR)-transformed relative abundance data without environmental features had the highest AUC (0.87, SD = 0.10) and second highest Kappa score (0.51, SD = 0.20) (Fig. 2; Table S2). When using genus-level data, the CF model built using the CLR-transformed relative abundance data without environmental features had the highest AUC (0.80, SD = 0.12) and Kappa (0.42, SD = 0.21) score. When using family-level data, the CF model using the relative abundance data with environmental features resulted in the highest Kappa score (0.34, SD = 0.23), and second highest AUC (0.76, SD = 0.14) (Fig. 2; Table S2). However, we found that the performance of models built using microbiome data only and that of models using microbiome and environmental factors were not significantly different when cross-validated performance measures were compared (paired *t* test, *P* > 0.05).

### The two most informative taxa for predicting Salmonella presence both belonged to the *Aeromonas* genus.

**FIG 3:**
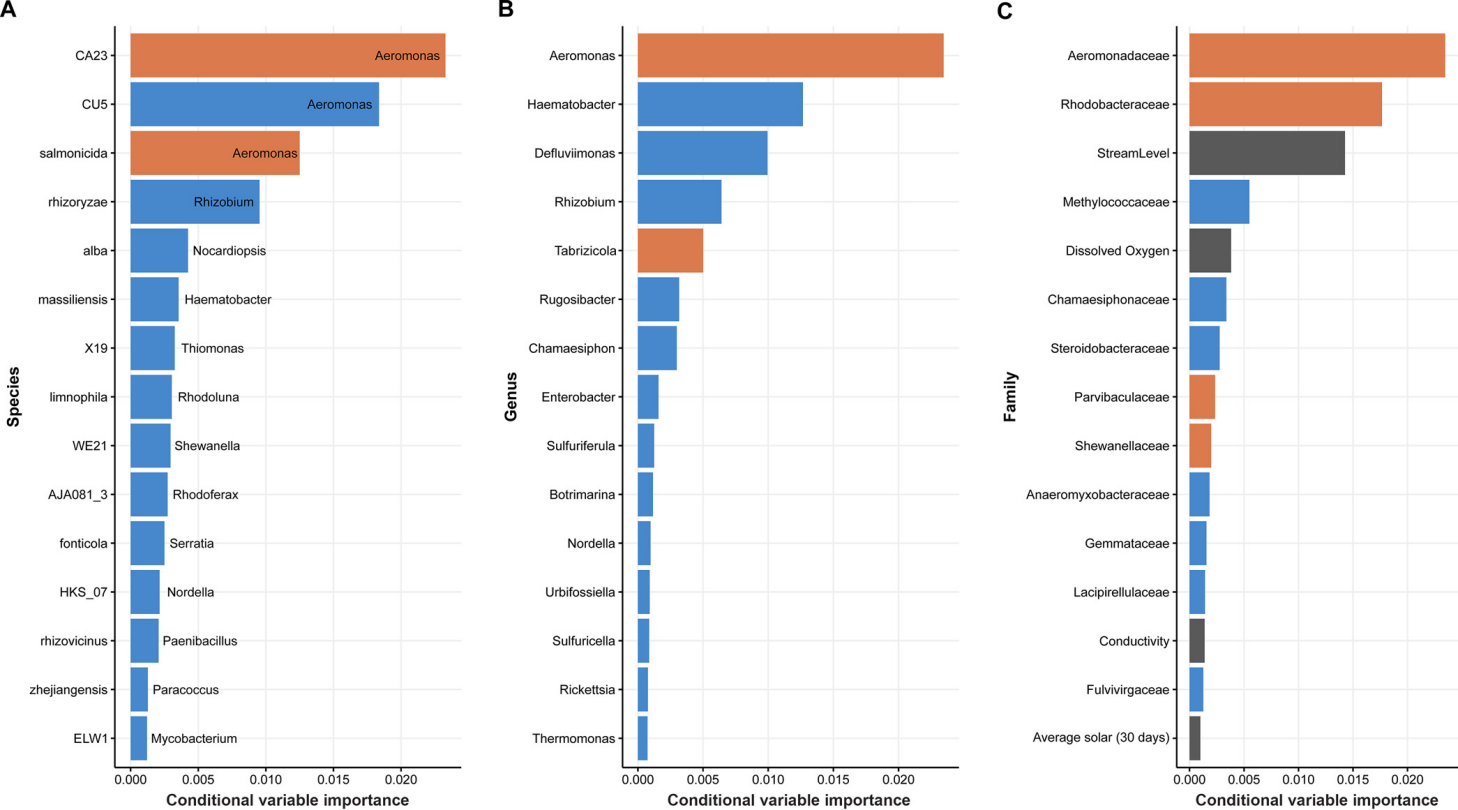
Conditional variable importance of taxa. Conditional variable importance was calculated from the best-performing conditional forest (CF) models built using species data (A), genus data (B), and family data (C). The top 15 most informative taxa for prediction of Salmonella contamination are presented. Orange bars indicate taxa significantly differentially abundant based on the differential abundance statistical test, blue bars indicate taxa not significantly differentially abundant, and black bars indicate environmental features.

CF identified *Aeromonas* sp. strain CA23, *Aeromonas* strain CU5, Aeromonas salmonicida, Rhizobium rhizoryzae, and Nocardiopsis alba as the five most informative species for predicting Salmonella contamination (Fig. 3A). Using genus-level microbiome data, CF identified *Aeromonas*, *Tabrizicola*, *Haematobacter*, *Defluviimonas*, and *Rhizobium* as the five most informative genera (Fig. 3B). In family-level analysis, *Aeromonadaceae*, *Rhodobacteraceae*, and *Methanobacteriaceae* were identified as the three most informative families. Furthermore, four environmental features (i.e., stream level, dissolved oxygen level, conductivity, and average amount of solar radiation in the last 30 days) were also identified as informative features in the CF model that included environmental features (Fig. 3C).

**FIG 4:**
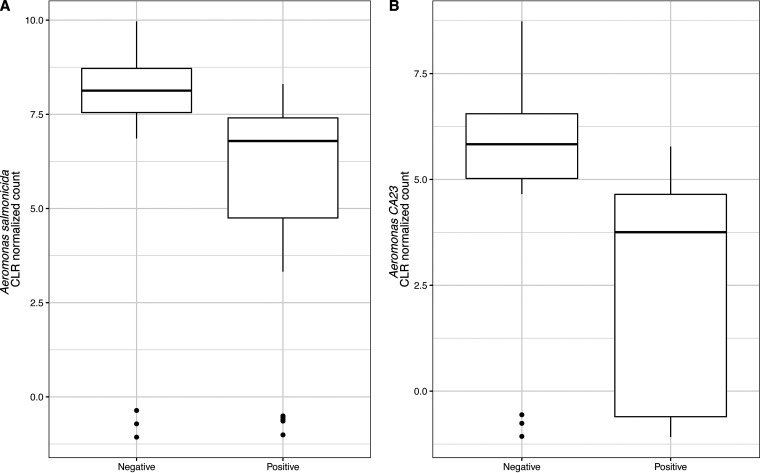
Relative abundance of Aeromonas salmonicida (A) and *Aeromonas* sp. CA23 (B) in water samples. These species were identified as significantly differentially abundant between Salmonella-positive and -negative water samples.

**FIG 5:**
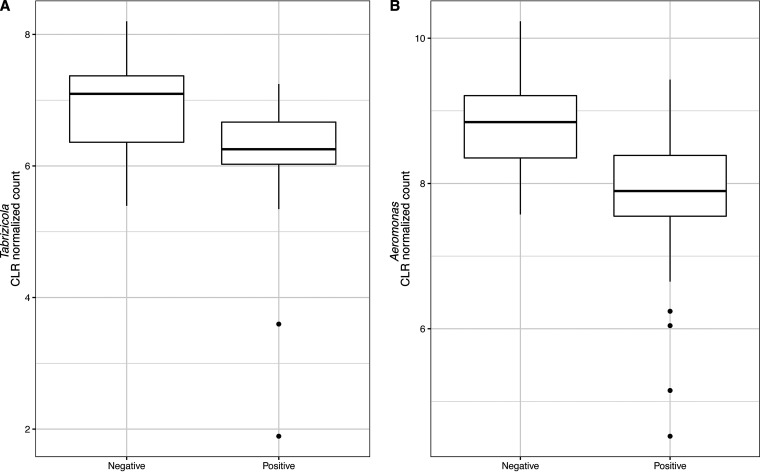
Relative abundance of *Tabrizicola* (A) and *Aeromonas* (B) in water samples. These genera were identified as significantly differentially abundant between Salmonella-positive and -negative water samples.

**FIG 6:**
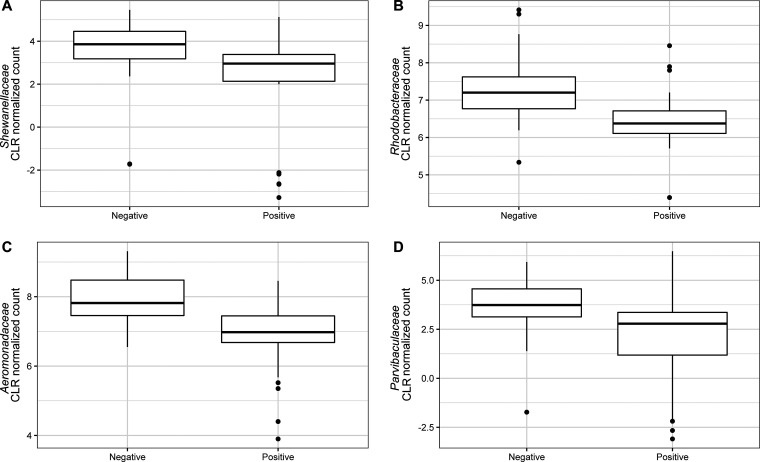
Relative abundance of *Shewanellaceae* (A), *Rhodobacteraceae* (B), *Parvibaculaceae* (C), and *Aeromonadaceae* (A). These families were identified as significantly differentially abundant between Salmonella-positive and -negative water samples.

The differential abundance analysis carried out using ALDEx2 identified two bacterial species, *Aeromonas* sp. CA23 (*P* = 0.00159) and Aeromonas salmonicida (*P* = 0.00172) (Fig. 4) as significantly differentially abundant between Salmonella-positive and Salmonella-negative samples. This was consistent with the results of the differential abundance test using genus-level microbiome data, where two bacterial genera, *Aeromonas* (*P* = 0.03246) and *Tabrizicola* (*P* = 0.037197) (Fig. 5) were identified as significantly differentially abundant between Salmonella-positive and Salmonella*-*negative samples. *Aeromonas* and *Tabrizicola* belong to the *Aeromonadaceae* and *Rhodobacteraceae* families, respectively. These two families are among the families identified as differentially abundant between Salmonella-positive and -negative samples (i.e., *Aeromonadaceae* [*P* = 0.01182], *Parvibaculaceae* [*P* = 0.045734], *Rhodobacteraceae* [*P* = 0.01058], and *Shewanellaceae* [*P* = 0.02342]) (Fig. 6).

## DISCUSSION

This study was based on the premise that contamination of surface waters with enteric pathogens such as Salmonella cooccurs with other, more abundant microbiota that are easier to rapidly and directly (e.g., by PCR without culture-based enrichment) detect. Such microbiota may be introduced to the waterway from the same contamination source as the target pathogen or be associated with the environmental conditions that favor pathogen introduction to, or survival in, freshwater systems. As such, these taxa could be useful as indicators of when and where surface waters may by contaminated by Salmonella and thus represent possible microbial targets of rapid tests used to monitor surface water for Salmonella contamination. Hence, we aimed to leverage water microbiome data and machine learning classifiers to identify specific taxa predictive of Salmonella contamination that could be further developed into rapid detection assays (10). For example, taxa that (i) consistently occur in samples contaminated with Salmonella or (ii) are consistently present in a significantly different relative abundance in samples contaminated with Salmonella may be utilized to develop rapid PCR-based indicator assays in preference over the current time-consuming enrichment-based detection method.

### Data transformation did not have a notable effect on the performance of predictive models.

Microbiome data analyses are challenging due to inherent data complexities. These include data sparsity (i.e., many taxa are present in only small proportions of samples, resulting in a large proportion of zero counts), collinearity (i.e., some taxa are highly correlated), imbalanced library sizes, and a well-known “small n, large p” problem (i.e., small number of samples and a large number of taxa). Some of these challenges can be addressed prior to applying machine learning models or can be addressed by applying machine learning methods that are robust to these microbiome data challenges. Here, we used a central log-ratio (CLR) transformation to mitigate the potential effects of different library sizes and to take into consideration the compositional nature of microbiome data (33, 34). We found that the microbiome data transformation had an inconsistent effect on a model performance, as it improved the AUC of some models (e.g., CF using genus- and family-level data and SVM using family-level data) while decreasing the AUC of other models (e.g., RRF using genus- and family-level data and SVM using genus-level data), whereas there were no significant differences among different models. Previous studies on gut microbiome data emphasized the importance of data transformation due to the compositionality of the microbiome data (28, 30, 32). However, similar to our finding, those studies also reported that the performance of tree-based algorithms (i.e., random forest and XGBoost) was not significantly affected by data transformation (32).

### Model selection is critically important when applying machine learning algorithms to microbiome data.

Different machine learning classifiers address microbiome data challenges to various degrees. Hence, we assessed the performance of three different machine learning algorithms for prediction of Salmonella contamination. At all taxonomic levels of microbiome data used for classification, we found that CF performed better overall than RRF and SVM. This is consistent with another study that compared the abilities of different algorithms to accurately predict foodborne pathogen contamination of surface water and consistently found that CF was a high-performing algorithm (27). However, in that study, the authors also found that RRF and/or SVM performed well. Given the complex relationships that underpin microbial ecosystems, algorithms that use hierarchical relationships between the presence-absence of various taxa (e.g., models that incorporate “interactions” or hierarchy such as CF and RRF) may predict pathogen presence better than algorithms that do not (e.g., SVM).

### Differential abundance analysis and conditional forest models identified putative indicators of Salmonella contamination in surface waters.

When assessing the association between the overall microbiome composition and Salmonella contamination, associations between certain taxa and Salmonella contamination may be missed due to the large number of taxa included in the analyses. Indeed, we found a lack of association between the overall microbiome composition and the presence of Salmonella based on the principal-component analysis (PCA) and PERMANOVA. Hence, we used differential abundance analysis and CF to identify individual taxa (or their relative abundance) associated with Salmonella contamination.

Both differential abundance analysis and CF identified some of the same taxa predictive of Salmonella contamination in stream water samples, suggesting that the association between Salmonella contamination and the presence of these taxa identified in the present study is robust to the analytical method used. Specifically, we identified two bacterial species (Aeromonas salmonicida and *Aeromonas* sp. CA23) which were present in a significantly lower relative abundance in samples contaminated with Salmonella using ALDEx2 differential abundance analysis. These taxa were also among the top-ranked factors based on the CF. *Aeromonas* species have previously been found in natural water and a broad range of foods, as well as human and animal gastrointestinal tracts (35, 36). *Aeromonas* sp. strain CA23 is closely related phylogenetically to *A. salmonicida* strain CBA100 (37). Aeromonas salmonicida has been found in a variety of aquatic environments and has been identified as a cause of disease in salmonid fish and wild cod (38, 39). *Aeromonas* has also been reported as an opportunistic pathogen in immunocompromised humans (35). However, the data on the relationship between *A. salmonicida* and Salmonella are limited. McIntosh et al. reported that antibiotic and mercury resistance genes were found on an *A. salmonicida* plasmid that is highly similar to pSN254, a plasmid previously identified in Salmonella (40). This may suggest horizontal gene transfer between the two genera, which requires cooccurrence in an environmental reservoir. Our study found that a higher relative abundance of *A. salmonicida* was associated with Salmonella-negative samples. This could possibly indicate that *A. salmonicida* is able to outcompete Salmonella, resulting in exclusion of Salmonella from the water microbiome or vice versa. Furthermore, *Aeromonas* has morphological and biochemical characteristics similar to those of microorganisms belonging to *Enterobacteriaceae*, a family of microorganisms that includes Salmonella and is commonly used as an indicator of poor hygienic conditions in food systems (41). Bonadonna et al. reported that the presence of E. coli and fecal coliforms was associated with lower counts of *Aeromonas*, whereas the prevalence of total coliforms (which include non-*Enterobacteriaceae*) was associated with higher counts of *Aeromonas* in bathing waters along the coast of the Adriatic Sea (42, 43). Due to limited information available, the ecological relationship between Salmonella and *Aeromonas* is poorly understood. Hence, more research is needed to characterize it and explain reported associations between these genera.

Another genus identified in this study, *Tabrizicola*, belongs to the family *Rhodobacteraceae*, which are usually found in aquatic environments, including lakes and wastewater treatment facilities (44–46). However, the ecological role of *Tabrizicola* is also poorly understood, which limits our ability to meaningfully interpret our identification of *Tabrizicola* as predictive of Salmonella contamination.

At a family level, we identified *Aeromonadaceae* (including *Aeromonas*), *Rhodobacteraceae* (including *Tabrizicola*), *Shewanellaceae*, and *Parvibaculaceae* as being strongly associated with accurately predicting Salmonella contamination in the CF analyses and being significantly differentially abundant between Salmonella-positive and -negative samples. All four identified families are known marine or aquatic microbiome members commonly found in natural waters (47–49). However, their relationship with Salmonella is unknown.

A study by Gu et al. used 16S rRNA gene amplicon sequencing data and found an association between specific microbial taxa and the prevalence and population density of Salmonella enterica detected in ponds and wells on the Eastern Shore of Virginia (ESV) between January and December (50). They found that the relative abundance of *Sphingomonadales* was significantly correlated with S. enterica prevalence and concentration in irrigation ponds and wells (50). However, in our study, we failed to find evidence of an association between *Sphingomonadales* and Salmonella contamination. This inconsistency could be explained by regional differences in the water microbiome composition or by differences in the microbiomes of ponds, wells, and streams. Similarly, microbiome composition is known to be influenced by environmental features such as natural variation over time, weather, and adjacent land use (22, 23, 25), and environmental differences between the waterways, water types, regions, or years may also explain the different findings.

Previous studies reported that environmental features had an effect on the probability of isolating Salmonella from freshwater samples (20, 50, 51). Therefore, it is important to consider environmental condition as supplementary features when developing new tools for predicting Salmonella contamination of freshwater based on the specific taxa. However, in our study, environmental features did not significantly improve the prediction accuracy.

### Conclusions.

This study applied machine learning classifiers and differential abundance analysis to surface water microbiome data to identify putative novel indicators of Salmonella contamination. We identified *Aeromonas* and *Tabrizicola* bacterial genera and *Aeromonadaceae*, *Rhodobacteraceae*, *Shewanellaceae*, and *Parvibaculaceae* families that warrant further assessment as putative indicators of Salmonella contamination of water from streams. The taxa identified here are potential targets for the development of an alternative or complementary (to E. coli quantification) water quality/safety monitoring strategy focused on mitigating the use of surface waters likely contaminated with Salmonella. Models developed in this study need to be validated on new samples collected from a broader geographic area and over multiple seasons to assess the predictive accuracy of taxa identified here. Furthermore, deeper metagenomic sequencing that would enable metagenome assembly may facilitate identification of putative novel indicators of Salmonella contamination as well as characterization of their functional potential to explain their ecological relationship with Salmonella.

## MATERIALS AND METHODS

### Sample collection and processing.

Water samples were collected from 60 streams in upstate New York State between July and October 2018 as described by Weller et al. (20). All chemical, microbial, and environmental water quality data were previously reported by Weller et al. (20). Briefly, 10-L grab samples were collected from each stream and tested for Salmonella presence. Each grab sample was filtered through modified Moore swabs (mMS). Buffered peptone water supplemented with novobiocin (20 mg/l) was added to Whirl-Pak bags containing mMS and incubated at 35°C for 24 h. After incubation, a BAX real-time PCR screen was used to identify samples that were presumptively positive for Salmonella. Salmonella presence was confirmed using culture-based methods fully described in the work of Weller et al. (20).

Separately, 100-mL grab samples were collected for metagenomic analysis. The 100-mL samples were filtered through a 0.45-mm filter (Nalgene; Thermo Fisher Scientific, Waltham, MA, USA). Filters were then stored at −80°C until DNA extraction.

### DNA extraction and microbiome sequencing.

DNA was extracted using the DNeasy Power Water kit (Qiagen, MD, USA) per the manufacturer’s instructions. Extracted DNA was examined for quality and quantified using Nanodrop One (Thermo Fisher Scientific, MA, USA) and Qubit 3 (Thermo Fisher Scientific, MA, USA), respectively. DNA was then sent to the Penn State Genomics Core Facility for library preparation and sequencing. Libraries were prepared using Nextera XT Flex per the manufacturer’s instructions. Pooled libraries were sequenced on an Illumina NextSeq with 150-bp paired-end reads.

### Sequence quality control and taxonomic classification.

FastQC version 0.11.5 was used to assess read quality using default parameters (52). Illumina adapters and low-quality bases were trimmed using Trimmomatic (v 0.36) (53) with default parameters. Trimmed reads were taxonomically classified using Kraken2 (v 2.1.2) (54), and abundances were inferred using Bracken (v 2.5) (55). The NCBI’s RefSeq nucleotide database (v 207) (56) was used to build a Kraken2 database. Any read that mapped to a single reference genome was labeled with the NCBI taxonomic annotation (taxid) corresponding to that reference genome. Any read that mapped to multiple reference genomes or did not meet or exceeded the confidence scoring threshold was assigned a last common ancestor (LCA) taxonomic identification (taxid) (54). Confidence scores were set to 0.1, meaning that at least 10% of the total number of kmers from a read were classified. Bracken was used to estimate the abundance of taxa by redistributing reads in the taxonomy using Bayes’ theorem (55). Assigned taxonomy and taxid counts of all samples were merged into a table that was used for downstream analyses.

### Microbiome analyses.

All statistical analyses of microbiome data were performed in R (version 4.1.2; R Core Team, Vienna, Austria) (57), using a compositional analyses framework (33). First, the estimated abundances were transformed using the centered log-ratio (CLR) transformation (34). Ratio transformations capture the relationship between the taxonomic units in the data, and the logarithm of these ratios ensures that the data are symmetric and linearly related (34). Distances between samples were calculated using the Aitchison distance (i.e., Euclidian distance after CLR transformation) to investigate the among-sample differences in compositional microbiome data (33). Principal-component analysis (PCA) was carried out using the ‘princomp’ function in R on relative abundances of taxids to visualize the ordination and clustering of samples based on the microbiome composition (33). The first two principal components were plotted using the ‘ggplot2’ package (v 3.3.3) (58). Samples were color coded to visually assess whether they cluster based on the Salmonella presence/absence. Permutational multivariate analysis of variance (PERMANOVA) was carried out to assess statistical associations between microbiome composition and Salmonella presence using the ‘adonis’ function in the ‘vegan’ package (v 2.5.7) (59). Differential abundance testing was conducted using the ALDEx2 R package (60) to identify bacterial species, genera, and families that were differentially abundant between Salmonella-positive and -negative samples. Each identified bacterial species, genus, and family were tested using Welch’s *t* test to assess statistical significance of detected differences in their relative abundance. To mitigate reporting false-positive results, we concurrently carried out differential abundance statistical tests and reported as putative novel indicators only taxa that were identified by both machine learning classifiers and ALDEx2.

### Predictive modeling.

Three machine learning algorithms (i.e., conditional forest [CF] [61], regularized random forest [RRF] [62], and support vector machine [SVM] with sigmoid kernel [63]) that had previously been applied on microbiome data (64–66) and were suitable for microbiome data structure (27) were used in this study. Additionally, these algorithms were previously reported to outperform others for predicting Salmonella presence using environmental data (27). These methods were used to develop models that predict Salmonella presence or absence in water samples. Both relative abundance of taxids and CLR-transformed relative abundance of taxids were separately used as features to assess the effect of microbiome data transformation on model performance. Model training and evaluation were performed using the ‘mlr’ package (v 2.19.0) (67). Tenfold cross-validation repeated 10 times was used to tune hyperparameters to maximize area under the curve (AUC) (68, 69). For the CF model, mtry and minbucket (number of taxa included in each splitting) were tuned. For the RRF model, mtry, nodesize (minimum node size of each decision tree), and coefReg (the coefficient of regularization) were tuned. Lastly, for SVM, cost and gamma were tuned to find the best-performing model (see Table S1 in the supplemental material). In total, analyses were conducted using two feature sets, (i) untransformed relative abundances (abundance of specific taxa/total count of taxids) of microbial taxa and (ii) CLR-transformed relative abundances of microbial taxa, to assess whether models perform better on transformed microbiome data. Analyses were also carried out at three different taxonomic levels (i.e., species, genus, and family) and with or without environmental data. Environmental features were provided from Table S1 in the work of Weller et al. (27). In total, 36 models were constructed separately based on the mean AUC and kappa scores (Table 1). The best-performing model for each combination of algorithm and input data (Table S2) was selected for identifying informative taxa. Paired *t* test was used assess the statistical significance of differences among selected models (70). Conditional variable importance (CVI) was calculated using the ‘party’ package (v. 1.3.7) for CF models (61). Conditional variable importance was used to mitigate the problem of complex interaction between bacterial taxa (i.e., highly correlated taxids) by screening correlated variables during the decision tree building process (61).

**TABLE 1:** Machine learning algorithms, data types, data transformation, and taxonomic levels used for prediction of Salmonella contamination

Machine learning algorithm^*a*^	Data type	Data transformation	Taxonomic level
Conditional forest	Microbiome data	Centered log-ratio-transformed relative abundances	Species
Regularized random forest	Microbiome data plus environmental features	Untransformed relative abundances	Genus
Support vector machine			Family

a Each machine learning algorithm was run using all combinations of data type, data transformation, and taxonomic level.

### Data availability.

Sequences generated in this study are available in the NCBI Sequence Read Archive database under the BioProject accession number PRJNA849616. Scripts used for bioinformatics and statistical analyses are available in the GitHub repository: https://github.com/tuc289/SurfaceWaterMicrobiome/tree/master/Year2.

## References

[1] Painter JA, Hoekstra RM, Ayers T, Tauxe RV, Braden CR, Angulo FJ, Griffin PM. 2013. Attribution of foodborne illnesses, hospitalizations, and deaths to food commodities by using outbreak data, United States, 1998–2008. Emerg Infect Dis 19:407–415. doi:10.3201/eid1903.111866. 23622497 PMC3647642

[2] Scallan E, Mahon BE. 2012. Foodborne Diseases Active Surveillance Network (FoodNet) in 2012: a foundation for food safety in the United States. Clin Infect Dis 54:S381–S384. doi:10.1093/cid/cis257. 22572657 PMC3348949

[3] Carstens CK, Salazar JK, Darkoh C. 2019. Multistate outbreaks of foodborne illness in the United States associated with fresh produce from 2010 to 2017. Front Microbiol 10:2667. doi:10.3389/fmicb.2019.02667. 31824454 PMC6883221

[4] US Food and Drug Administration. 2020. Factors potentially contributing to the contamination of romaine lettuce implicated in the three outbreaks of E. coli O157:H7 during the fall of 2019. US Food and Drug Administration, Silver Spring, MD.

[5] Centers for Disease Control and Prevention. 2019. Outbreak of E. coli infections linked to romaine lettuce. Centers for Disease Control and Prevention, Atlanta, GA.

[6] Weller D, Wiedmann M, Strawn LK. 2015. Irrigation is significantly associated with an increased prevalence of Listeria monocytogenes in produce production environments in New York State. J Food Prot 78:1132–1141. doi:10.4315/0362-028X.JFP-14-584. 26038903

[7] Strawn LK, Gröhn YT, Warchocki S, Worobo RW, Bihn EA, Wiedmann M. 2013. Risk factors associated with *Salmonella* and *Listeria monocytogenes* contamination of produce fields. Appl Environ Microbiol 79:7618–7627. doi:10.1128/AEM.02831-13. 24077713 PMC3837806

[8] European Parliament. 2006. Directive 2006/7/EC of the European Parliament and of the Council of 15 February 2006 concerning the management of bathing water quality and repealing Directive 76/160/EEC. 32006L0007064. European Parliament, Brussels, Belgium.

[9] EPA. 2012. 2012 recreational water quality criteria. https://www.epa.gov/sites/default/files/2015-10/documents/rwqc2012.pdf.

[10] FDA. 2021. FSMA final rule on produce safety. https://www.fda.gov/food/food-safety-modernization-act-fsma/fsma-final-rule-produce-safety. Retrieved 10 January 2022.

[11] Leafy Green Products Handler Marketing Agreement (LGMA). 2017. Commodity specific food safety guidelines for the production and harvest of lettuce and leafy greens. Arizona Department of Agriculture, Phoenix, AZ.

[12] Edberg S, Rice E, Karlin R, Allen M. 2000. Escherichia coli: the best biological drinking water indicator for public health protection. J Appl Microbiol 88:106S–116S. doi:10.1111/j.1365-2672.2000.tb05338.x. 10880185

[13] Wilkes G, Edge T, Gannon V, Jokinen C, Lyautey E, Medeiros D, Neumann N, Ruecker N, Topp E, Lapen DR. 2009. Seasonal relationships among indicator bacteria, pathogenic bacteria, Cryptosporidium oocysts, Giardia cysts, and hydrological indices for surface waters within an agricultural landscape. Water Res 43:2209–2223. doi:10.1016/j.watres.2009.01.033. 19339033

[14] Bihn E. 2011. Survey of current water use practices on fresh fruit and vegetable farms and evaluation of microbiological quality of surface waters intended for fresh produce production. PhD dissertation. Cornell University, Ithaca, NY.

[15] Payment P, Locas A. 2011. Pathogens in water: value and limits of correlation with microbial indicators. Groundwater 49:4–11. doi:10.1111/j.1745-6584.2010.00710.x. 20477877

[16] Benjamin L, Atwill ER, Jay-Russell M, Cooley M, Carychao D, Gorski L, Mandrell RE. 2013. Occurrence of generic Escherichia coli, E. coli O157 and Salmonella spp. in water and sediment from leafy green produce farms and streams on the Central California coast. Int J Food Microbiol 165:65–76. doi:10.1016/j.ijfoodmicro.2013.04.003. 23697918

[17] McEgan R, Mootian G, Goodridge LD, Schaffner DW, Danyluk MD. 2013. Predicting *Salmonella* populations from biological, chemical, and physical indicators in Florida surface waters. Appl Environ Microbiol 79:4094–4105. doi:10.1128/AEM.00777-13. 23624476 PMC3697547

[18] Antaki EM, Vellidis G, Harris C, Aminabadi P, Levy K, Jay-Russell MT. 2016. Low concentration of Salmonella enterica and generic Escherichia coli in farm ponds and irrigation distribution systems used for mixed produce production in southern Georgia. Foodborne Pathog Dis 13:551–558. doi:10.1089/fpd.2016.2117. 27400147 PMC6445212

[19] Pachepsky Y, Shelton D, Dorner S, Whelan G. 2016. Can E. coli or thermotolerant coliform concentrations predict pathogen presence or prevalence in irrigation waters? Crit Rev Microbiol 42:384–393. doi:10.3109/1040841X.2014.954524. 25198779

[20] Weller D, Brassill N, Rock C, Ivanek R, Mudrak E, Roof S, Ganda E, Wiedmann M. 2020. Complex interactions between weather, and microbial and physicochemical water quality impact the likelihood of detecting foodborne pathogens in agricultural water. Front Microbiol 11:134. doi:10.3389/fmicb.2020.00134. 32117154 PMC7015975

[21] Luo Z, Gu G, Ginn A, Giurcanu MC, Adams P, Vellidis G, van Bruggen AH, Danyluk MD, Wright AC. 2015. Distribution and characterization of *Salmonella enterica* isolates from irrigation ponds in the southeastern United States. Appl Environ Microbiol 81:4376–4387. doi:10.1128/AEM.04086-14. 25911476 PMC4475880

[22] Van Rossum T, Peabody MA, Uyaguari-Diaz MI, Cronin KI, Chan M, Slobodan JR, Nesbitt MJ, Suttle CA, Hsiao WWL, Tang PKC, Prystajecky NA, Brinkman FSL. 2015. Year-long metagenomic study of river microbiomes across land use and water quality. Front Microbiol 6:1405. doi:10.3389/fmicb.2015.01405. 26733955 PMC4681185

[23] Wang L, Zhang J, Li H, Yang H, Peng C, Peng Z, Lu L. 2018. Shift in the microbial community composition of surface water and sediment along an urban river. Sci Total Environ 627:600–612. doi:10.1016/j.scitotenv.2018.01.203. 29426184

[24] Payne JT, Millar JJ, Jackson CR, Ochs CA. 2017. Patterns of variation in diversity of the Mississippi river microbiome over 1,300 kilometers. PLoS One 12:e0174890. doi:10.1371/journal.pone.0174890. 28350888 PMC5370145

[25] Chung T, Weller DL, Kovac J. 2020. The composition of microbial communities in six streams, and its association with environmental conditions, and foodborne pathogen isolation. Front Microbiol 11:1757. doi:10.3389/fmicb.2020.01757. 32849385 PMC7403445

[26] Polat H, Topalcengiz Z, Danyluk MD. 2020. Prediction of Salmonella presence and absence in agricultural surface waters by artificial intelligence approaches. J Food Saf 40:e12733. doi:10.1111/jfs.12733.

[27] Weller DL, Love TM, Belias A, Wiedmann M. 2020. Predictive models may complement or provide an alternative to existing strategies for assessing the enteric pathogen contamination status of northeastern streams used to provide water for produce production. Front Sustain Food Syst 4:561517. doi:10.3389/fsufs.2020.561517. 33791594 PMC8009603

[28] Song K, Wright FA, Zhou Y-H. 2020. Systematic comparisons for composition profiles, taxonomic levels, and machine learning methods for microbiome-based disease prediction. Front Mol Biosci 7:610845. doi:10.3389/fmolb.2020.610845. 33392266 PMC7772236

[29] Marcos-Zambrano LJ, Karaduzovic-Hadziabdic K, Loncar Turukalo T, Przymus P, Trajkovik V, Aasmets O, Berland M, Gruca A, Hasic J, Hron K, Klammsteiner T, Kolev M, Lahti L, Lopes MB, Moreno V, Naskinova I, Org E, Paciência I, Papoutsoglou G, Shigdel R, Stres B, Vilne B, Yousef M, Zdravevski E, Tsamardinos I, Carrillo de Santa Pau E, Claesson MJ, Moreno-Indias I, Truu J. 2021. Applications of machine learning in human microbiome studies: a review on feature selection, biomarker identification, disease prediction and treatment. Front Microbiol 12:634511. doi:10.3389/fmicb.2021.634511. 33737920 PMC7962872

[30] Topçuoğlu BD, Lesniak NA, Ruffin IVMT, Wiens J, Schloss PD. 2020. A framework for effective application of machine learning to microbiome-based classification problems. mBio 11:e00434-20. doi:10.1128/mBio.00434-20. 32518182 PMC7373189

[31] Ghannam RB, Techtmann SM. 2021. Machine learning applications in microbial ecology, human microbiome studies, and environmental monitoring. Comput Struct Biotechnol J 19:1092–1107. doi:10.1016/j.csbj.2021.01.028. 33680353 PMC7892807

[32] Kubinski R, Djamen-Kepaou J-Y, Zhanabaev T, Hernandez-Garcia A, Bauer S, Hildebrand F, Korcsmaros T, Karam S, Jantchou P, Kafi K, Martin RD. 2022. Benchmark of data processing methods and machine learning models for gut microbiome-based diagnosis of inflammatory bowel disease. Front Genet 13:784397. doi:10.3389/fgene.2022.784397. 35251123 PMC8895431

[33] Gloor GB, Macklaim JM, Pawlowsky-Glahn V, Egozcue JJ. 2017. Microbiome datasets are compositional: and this is not optional. Front Microbiol 8:2224. doi:10.3389/fmicb.2017.02224. 29187837 PMC5695134

[34] Aitchison J, Barceló-Vidal C, Martín-Fernández JA, Pawlowsky-Glahn V. 2000. Logratio analysis and compositional distance. Math Geol 32:271–275. doi:10.1023/A:1007529726302.

[35] Altwegg M, Geiss HK, Freij BJ. 1989. Aeromonas as a human pathogen. CRC Crit Rev Microbiol 16:253–286. doi:10.3109/10408418909105478. 2649316

[36] Janda JM, Abbott SL. 2010. The genus Aeromonas: taxonomy, pathogenicity, and infection. Clin Microbiol Rev 23:35–73. doi:10.1128/CMR.00039-09. 20065325 PMC2806660

[37] Kooli WM, Junier T, Shakya M, Monachon M, Davenport KW, Vaideeswaran K, Vernudachi A, Marozau I, Monrouzeau T, Gleasner CD, McMurry K, Lienhard R, Rufener L, Perret JL, Sereda O, Chain PS, Joseph E, Junier P. 2019. Remedial treatment of corroded iron objects by environmental *Aeromonas* isolates. Appl Environ Microbiol 85:e02042-18. doi:10.1128/AEM.02042-18. 30478230 PMC6344634

[38] Soto-Dávila M, Hossain A, Chakraborty S, Rise ML, Santander J. 2019. *Aeromonas salmonicida* subsp. *salmonicida* early infection and immune response of Atlantic cod (*Gadus morhua* L.) primary macrophages. Front Immunol 10:1237. doi:10.3389/fimmu.2019.01237. 31231379 PMC6559310

[39] Dallaire-Dufresne S, Tanaka KH, Trudel MV, Lafaille A, Charette SJ. 2014. Virulence, genomic features, and plasticity of Aeromonas salmonicida subsp. salmonicida, the causative agent of fish furunculosis. Vet Microbiol 169:1–7. doi:10.1016/j.vetmic.2013.06.025. 23890675

[40] McIntosh D, Cunningham M, Ji B, Fekete FA, Parry EM, Clark SE, Zalinger ZB, Gilg IC, Danner GR, Johnson KA, Beattie M, Ritchie R. 2008. Transferable, multiple antibiotic and mercury resistance in Atlantic Canadian isolates of Aeromonas salmonicida subsp. salmonicida is associated with carriage of an IncA/C plasmid similar to the Salmonella enterica plasmid pSN254. J Antimicrob Chemother 61:1221–1228. doi:10.1093/jac/dkn123. 18375380 PMC2902851

[41] Zeitoun A, Debevere J, Mossel D. 1994. Significance of Enterobacteriaceae as index organisms for hygiene on fresh untreated poultry, poultry treated with lactic acid and poultry stored in a modified atmosphere. Food Microbiol 11:169–176. doi:10.1006/fmic.1994.1020.

[42] Burke V, Robinson J, Gracey M, Peterson D, Partridge K. 1984. Isolation of *Aeromonas hydrophila* from a metropolitan water supply: seasonal correlation with clinical isolates. Appl Environ Microbiol 48:361–366. doi:10.1128/aem.48.2.361-366.1984. 6385848 PMC241518

[43] Bonadonna L, Briancesco R, Coccia AM, Semproni M, Stewardson D. 2002. Occurrence of potential bacterial pathogens in coastal areas of the Adriatic Sea. Environ Monit Assess 77:31–49. doi:10.1023/a:1015734015382. 12139074

[44] Ko D-J, Kim J-S, Park D-S, Lee D-H, Heo S-Y, Seo J-W, Kim CH, Oh B-R. 2018. Tabrizicola fusiformis sp. nov., isolated from an industrial wastewater treatment plant. Int J Syst Evol Microbiol 68:1800–1805. doi:10.1099/ijsem.0.002760. 29624160

[45] Liu Z-X, Dorji P, Liu H-C, Li A-H, Zhou Y-G. 2019. Tabrizicola sediminis sp. nov., one aerobic anoxygenic photoheterotrophic bacteria from sediment of saline lake. Int J Syst Evol Microbiol 69:2565–2570. doi:10.1099/ijsem.0.003542. 31219417

[46] Tarhriz V, Eyvazi S, Shakeri E, Hejazi MS, Dilmaghani A. 2020. Antibacterial and antifungal activity of novel freshwater bacterium Tabrizicola aquatica as a prominent natural antibiotic available in Qurugol Lake. Pharm Sci 26:88–92. doi:10.34172/PS.2019.56.

[47] Esteve C. 1995. Numerical taxonomy of Aeromonadaceae and Vibrionaceae associated with reared fish and surrounding fresh and brackish water. Syst Appl Microbiol 18:391–402. doi:10.1016/S0723-2020(11)80432-7.

[48] Elifantz H, Horn G, Ayon M, Cohen Y, Minz D. 2013. Rhodobacteraceae are the key members of the microbial community of the initial biofilm formed in Eastern Mediterranean coastal seawater. FEMS Microbiol Ecol 85:348–357. doi:10.1111/1574-6941.12122. 23551015

[49] Satomi M. 2014. The family Shewanellaceae, p 597–625. *In* Rosenberg E, DeLong EF, Lory S, Stackebrandt E, Thompson F (ed), The prokaryotes. Gammaproteobacteria, 4th ed. Springer Verlag, Berlin, Germany.

[50] Gu G, Strawn LK, Ottesen AR, Ramachandran P, Reed EA, Zheng J, Boyer RR, Rideout SL. 2021. Correlation of *Salmonella enterica* and *Listeria monocytogenes* in irrigation water to environmental factors, fecal indicators, and bacterial communities. Front Microbiol 11:557289. doi:10.3389/fmicb.2020.557289. 33488530 PMC7820387

[51] Toro M, Weller D, Ramos R, Diaz L, Alvarez FP, Reyes-Jara A, Moreno-Switt AI, Meng J, Adell AD. 2022. Environmental and anthropogenic factors associated with the likelihood of detecting Salmonella in agricultural watersheds. Environ Pollut 306:119298. doi:10.1016/j.envpol.2022.119298. 35430308

[52] Andrews S. 2010. FastQC: a quality control tool for high throughput sequence data.

[53] Bolger AM, Lohse M, Usadel B. 2014. Trimmomatic: a flexible trimmer for Illumina sequence data. Bioinformatics 30:2114–2120. doi:10.1093/bioinformatics/btu170. 24695404 PMC4103590

[54] Wood DE, Lu J, Langmead B. 2019. Improved metagenomic analysis with Kraken 2. Genome Biol 20:257. doi:10.1186/s13059-019-1891-0. 31779668 PMC6883579

[55] Lu J, Breitwieser FP, Thielen P, Salzberg SL. 2017. Bracken: estimating species abundance in metagenomics data. PeerJ Comput Sci 3:e104. doi:10.7717/peerj-cs.104. PMC12016282 40271438

[56] Pruitt KD, Tatusova T, Maglott DR. 2007. NCBI reference sequences (RefSeq): a curated non-redundant sequence database of genomes, transcripts and proteins. Nucleic Acids Res 35:D61–D65. doi:10.1093/nar/gkl842. 17130148 PMC1716718

[57] R Core Team. 2020. R: a language and environment for statistical computing. R Foundation for Statistical Computing, Vienna, Austria. https://www.R-project.org/.

[58] Wickham H. 2011. ggplot2. WIREs Comp Stat 3:180–185. doi:10.1002/wics.147.

[59] Dixon P. 2003. VEGAN, a package of R functions for community ecology. J Veg Sci 14:927–930. doi:10.1111/j.1654-1103.2003.tb02228.x.

[60] Fernandes DA, Reid J, Macklaim MJ, McMurrough TA, Edgell DR, Gloor BG. 2014. Unifying the analysis of high-throughput sequencing datasets: characterizing RNA-seq, 16S rRNA gene sequencing and selective growth experiments by compositional data analysis. Microbiome 2:15. doi:10.1186/2049-2618-2-15. 24910773 PMC4030730

[61] Strobl C, Boulesteix A-L, Kneib T, Augustin T, Zeileis A. 2008. Conditional variable importance for random forests. BMC Bioinformatics 9:307. doi:10.1186/1471-2105-9-307. 18620558 PMC2491635

[62] Deng H, Runger G. 2013. Gene selection with guided regularized random forest. Pattern Recognit 46:3483–3489. doi:10.1016/j.patcog.2013.05.018.

[63] Cortes C, Vapnik V. 1995. Support-vector networks. Mach Learn 20:273–297. doi:10.1007/BF00994018.

[64] Moreno-Indias I, Lahti L, Nedyalkova M, Elbere I, Roshchupkin G, Adilovic M, Aydemir O, Bakir-Gungor B, Carrillo-de Santa Pau E, D’Elia D, Desai MS, Falquet L, Gundogdu A, Hron K, Klammsteiner T, Lopes MB, Marcos-Zambrano LJ, Marques C, Mason M, May P, Pašić L, Pio G, Pongor S, Promponas VJ, Przymus P, Saez-Rodriguez J, Sampri A, Shigdel R, Stres B, Suharoschi R, Truu J, Truică C-O, Vilne B, Vlachakis D, Yilmaz E, Zeller G, Zomer AL, Gómez-Cabrero D, Claesson MJ. 2021. Statistical and machine learning techniques in human microbiome studies: contemporary challenges and solutions. Front Microbiol 12:635781. doi:10.3389/fmicb.2021.635781. 33692771 PMC7937616

[65] Namkung J. 2020. Machine learning methods for microbiome studies. J Microbiol 58:206–216. doi:10.1007/s12275-020-0066-8. 32108316

[66] Zhou Y-H, Gallins P. 2019. A review and tutorial of machine learning methods for microbiome host trait prediction. Front Genet 10:579. doi:10.3389/fgene.2019.00579. 31293616 PMC6603228

[67] Bischl B, Lang M, Kotthoff L, Schiffner J, Richter J, Studerus E, Casalicchio G, Jones ZM. 2016. mlr: Machine Learning in R. J Mach Learn Res 17:1–5.

[68] Kim J-H. 2009. Estimating classification error rate: repeated cross-validation, repeated hold-out and bootstrap. Comput Stat Data Anal 53:3735–3745. doi:10.1016/j.csda.2009.04.009.

[69] Hanley JA, McNeil BJ. 1982. The meaning and use of the area under a receiver operating characteristic (ROC) curve. Radiology 143:29–36. doi:10.1148/radiology.143.1.7063747. 7063747

[70] Hsu H, Lachenbruch PA. 2014. Paired t test. Wiley StatsRef Statistics Reference Online. Wiley, Hoboken, NJ.

