# Supplementary material for: Conditional Forest Models Built Using Metagenomic Data Accurately Predicted Salmonella Contamination in Northeastern Streams
Source: Microbiol Spectr. 2023 Mar 22;11(2):e00381-23. doi: 10.1128/spectrum.00381-23 (PMC10100987; doi:10.1128/spectrum.00381-23)
Supplement: Supplemental file 1 — Supplemental material. Download spectrum.00381-23-s0001.pdf, PDF file, 1.7 MB [file spectrum.00381-23-s0001.pdf]

**SUPPLEMENTAL MATERIAL**

# Conditional forest models built using metagenomic data accurately predicted *Salmonella* contamination in Northeastern streams

Taejung Chung[1,2], Runan Yan[1,2], Daniel L. Weller[3], Jasna Kovac[1,2]

[1] Department of Food Science, The Pennsylvania State University, University Park, Pennsylvania, USA
[2] Microbiome Center, Huck Institutes of the Life Sciences, The Pennsylvania State University, University Park, Pennsylvania, USA
[3] Department of Statistics and Computational Biology, University of Rochester Medical Center, Rochester, NY, USA

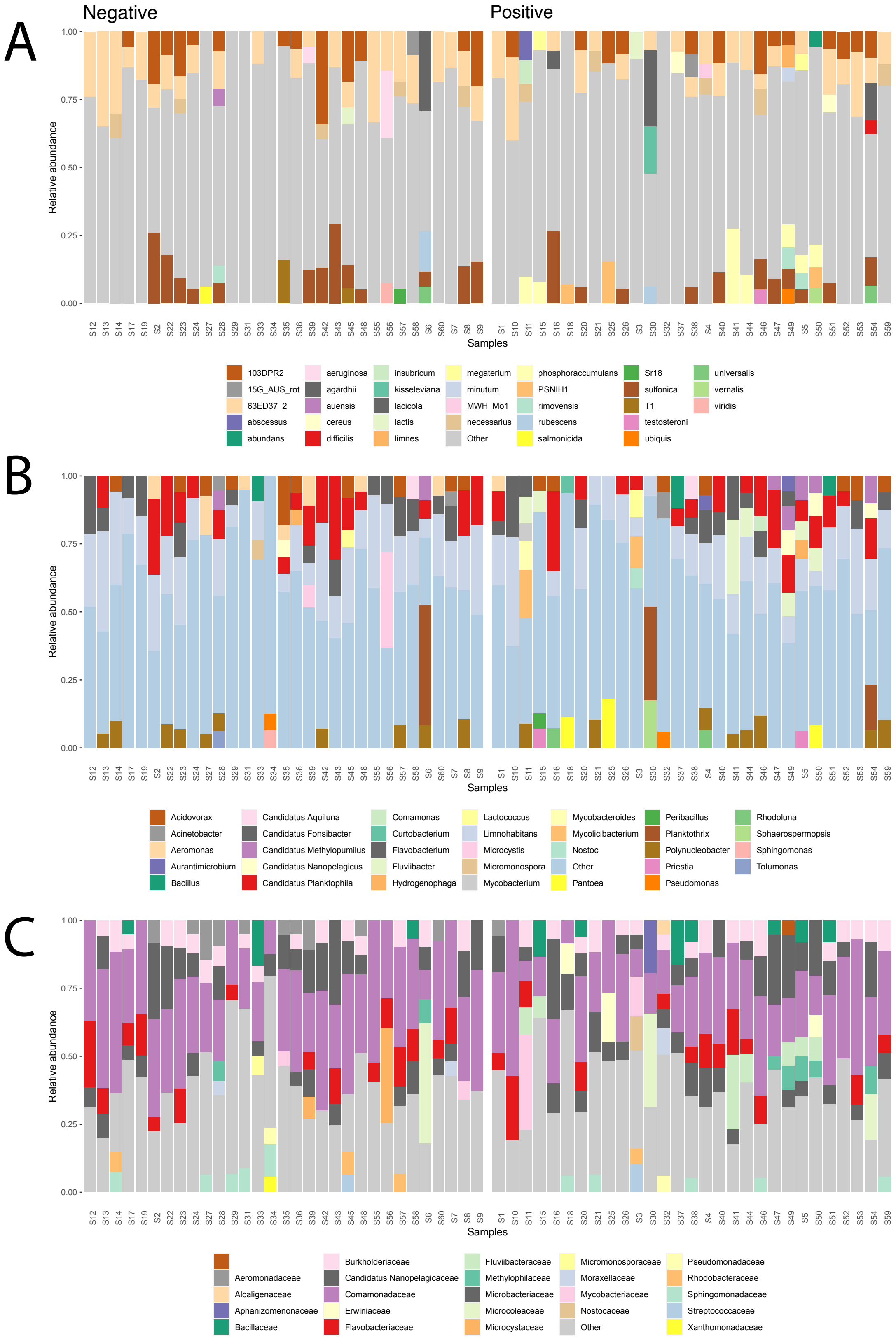


**FIG S1** Microbiome composition of water samples at a (A) species, (B) genus, and (C) family level.

**Table S1** Tuned hyper parameters for each classification model.

| Level of taxa | Data type[a] | Data Transformation[b] | **cforest** | | | | **RRF** | | | | **SVM** | | |
|---|---|---|---|---|---|---|---|---|---|---|---|---|---|
| | | | ntree | mtry | minbucket | mincriterion | ntree | mtry | nodesize | coefReg | kernel | cost | gamma |
| Species | M | CLR | 5001 | 658 | 15 | 0.95 | 5001 | 58 | 25 | 1 | sigmoid | 100 | 0.1 |
| Species | M | REL | 5001 | 656 | 15 | 0.95 | 5001 | 58 | 23 | 1 | sigmoid | 10 | 10 |
| Species | M + E | CLR | 5001 | 756 | 15 | 0.95 | 5001 | 56 | 36 | 1 | sigmoid | 1 | 10 |
| Species | M + E | REL | 5001 | 352 | 13 | 0.95 | 5001 | 2406 | 27 | 1 | sigmoid | 10 | 10 |
| Genus | M | CLR | 5001 | 8 | 8 | 0.95 | 5001 | 60 | 24 | 1 | sigmoid | 100 | 1 |
| Genus | M | REL | 5001 | 152 | 3 | 0.95 | 5001 | 1254 | 14 | 1 | sigmoid | 0.01 | 0.1 |
| Genus | M + E | CLR | 5001 | 104 | 13 | 0.95 | 5001 | 60 | 25 | 1 | sigmoid | 1000 | 0.1 |
| Genus | M + E | REL | 5001 | 4 | 9 | 0.95 | 5001 | 1908 | 37 | 1 | sigmoid | 10 | 100 |
| Family | M | CLR | 5001 | 54 | 3 | 0.95 | 5001 | 56 | 36 | 0.95 | sigmoid | 0.001 | 1 |
| Family | M | REL | 5001 | 208 | 11 | 0.95 | 5001 | 152 | 4 | 0.95 | sigmoid | 1 | 1 |
| Family | M + E | CLR | 5001 | 4 | 1 | 0.95 | 5001 | 54 | 34 | 0.95 | sigmoid | 100 | 1 |
| Family | M + E | REL | 5001 | 152 | 15 | 0.95 | 5001 | 500 | 36 | 0.95 | sigmoid | 1000 | 1 |

[a] M, microbiome data; E, environmental data.

[b] CLR, central log-ratio transformation; REL, relative abundance.

**Table S2** Model performance based on 10 times 10-fold repeated cross validation.

| Model | Taxonomic level | Data type[a] | Data transformation[b] | Mean AUC[c] | Mean Kappa | AUC SD | Kappa SD |
|---|---|---|---|---|---|---|---|
| cForest | Species | M | CLR | 0.87 | 0.51 | 0.10 | 0.20 |
| cForest | Species | M + E | CLR | 0.86 | 0.53 | 0.11 | 0.25 |
| cForest | Species | M | REL | 0.83 | 0.46 | 0.14 | 0.23 |
| cForest | Species | M + E | REL | 0.83 | 0.45 | 0.11 | 0.18 |
| cForest | Genus | M | CLR | 0.81 | 0.42 | 0.12 | 0.21 |
| cForest | Genus | M + E | CLR | 0.80 | 0.38 | 0.13 | 0.20 |
| cForest | Family | M + E | REL | 0.76 | 0.34 | 0.14 | 0.23 |
| cForest | Family | M | REL | 0.75 | 0.33 | 0.14 | 0.22 |
| cForest | Genus | M + E | REL | 0.74 | 0.30 | 0.17 | 0.30 |
| cForest | Genus | M | REL | 0.73 | 0.28 | 0.16 | 0.28 |
| cForest | Family | M + E | CLR | 0.72 | 0.27 | 0.14 | 0.26 |
| cForest | Family | M | CLR | 0.67 | 0.17 | 0.15 | 0.28 |
| RRF | Species | M | CLR | 0.81 | 0.41 | 0.13 | 0.26 |
| RRF | Species | M | REL | 0.80 | 0.36 | 0.10 | 0.20 |
| RRF | Species | M + E | CLR | 0.80 | 0.37 | 0.14 | 0.22 |
| RRF | Genus | M + E | CLR | 0.78 | 0.34 | 0.11 | 0.21 |
| RRF | Species | M + E | REL | 0.77 | 0.35 | 0.16 | 0.30 |
| RRF | Genus | M | REL | 0.76 | 0.33 | 0.14 | 0.23 |
| RRF | Family | M + E | CLR | 0.75 | 0.34 | 0.14 | 0.23 |
| RRF | Family | M | CLR | 0.75 | 0.34 | 0.17 | 0.27 |
| RRF | Family | M | REL | 0.74 | 0.24 | 0.14 | 0.22 |
| RRF | Family | M + E | REL | 0.73 | 0.11 | 0.14 | 0.18 |
| RRF | Genus | M + E | REL | 0.73 | 0.06 | 0.14 | 0.18 |
| RRF | Genus | M | CLR | 0.71 | 0.30 | 0.16 | 0.26 |
| SVM | Species | M | REL | 0.65 | 0.12 | 0.17 | 0.23 |
| SVM | Species | M | CLR | 0.65 | 0.11 | 0.20 | 0.23 |
| SVM | Family | M | REL | 0.65 | 0.27 | 0.16 | 0.21 |
| SVM | Genus | M | CLR | 0.61 | 0.11 | 0.20 | 0.23 |
| SVM | Genus | M | REL | 0.60 | 0.12 | 0.17 | 0.23 |
| SVM | Species | M + E | CLR | 0.58 | 0.01 | 0.17 | 0.24 |
| SVM | Species | M + E | REL | 0.54 | 0.00 | 0.00 | 0.00 |
| SVM | Family | M + E | REL | 0.51 | 0.00 | 0.00 | 0.00 |
| SVM | Genus | M + E | REL | 0.50 | 0.00 | 0.00 | 0.00 |
| SVM | Family | M + E | CLR | 0.48 | 0.01 | 0.19 | 0.24 |
| SVM | Genus | M + E | CLR | 0.48 | 0.01 | 0.17 | 0.24 |
| SVM | Family | M | CLR | 0.44 | 0.08 | 0.14 | 0.20 |

[a] M, microbiome data; E, environmental data.
[b] CLR, central log-ratio transformation; REL, relative abundance.
[c] AUC, area under the receiver operating characteristic curve.
